# DoRes within CellMissy: dose-response analysis on cell migration and related data

**DOI:** 10.1093/bioinformatics/bty634

**Published:** 2018-07-25

**Authors:** Gwendolien Sergeant, Lennart Martens, Marleen Van Troys, Paola Masuzzo

**Affiliations:** 1 VIB-UGent Center for Medical Biotechnology, Ghent, Belgium; 2 Department of Biochemistry, Faculty of Medicine and Health Sciences, Ghent University, Ghent, Belgium

## Abstract

**Summary:**

In cancer research, cell-based assays are used to assess cell migration and invasion. The major bottleneck is the lack of automated tools to visualize and analyse the large amounts of biological dose-response data produced. To address this challenge, we have developed an automated and free software package for dose-response analyses, DoRes, which is released as an add-on of the freely available and open-source tool CellMissy, dedicated to the management and analysis of cell migration data. DoRes implements non-linear curve fitting functionality into a robust, user-friendly and flexible software package with the possibility of importing a tabular file or starting from a cell migration experiment. We demonstrate the ability of the software by analysing public dose-response data and a typical cell migration experiment, and show that the extracted dose-response parameters and the calculated statistical values are consistently comparable to those of the widely used, commercial software GraphPad Prism.

**Availability and implementation:**

The software here presented is a new module in CellMissy, an open-source and cross-platform package dedicated to the management, storage and analysis of cell migration data. The new module is written in Java, and inherits the cross-platform support from CellMissy. Source code and binaries are freely available under the Apache2 open-source licence at https://github.com/compomics/cellmissy/.

**Supplementary information:**

Supplementary data are available at *Bioinformatics* online.

## 1 Introduction

*In vitro* cell-based assays are often used to test how candidate components or treatments influence certain cellular functions. Effects on cell proliferation, adhesion, migration, invasion, cell apoptosis, morphology changes and more can be measured (Khalili and Ahmad, 2015; Kramer *et al.*, 2013). When used in high-throughput fashion, these *in vitro* assays play a crucial role in the identification of biological agents and comparison of their potency (Nierode *et al.*, 2016). An important type of study is a dose-response experiment, in which a specific compound is applied in parallel in different concentrations, and the relationship between the concentration of this compound to the response of the biological system under study is quantified, usually to determine the optimal dose of the compound (Lopez Jornet *et al.*, 2015). The datasets obtained through such high-throughput dose-response experiments are often large and contain information on different levels, which necessitates data storage and automated analysis tools (Masuzzo *et al.*, 2016). These analysis tools speed up processing time and reduce the possibility for human error. Moreover, when connected directly to a data storage solution, these tools also allow meta-analyses to take place. For high-throughput dose-response experiments, these analytical tools need to be able to quickly and accurately estimate the dose-response curve, even for very large datasets and in the case of high variance. This curve estimation is typically achieved by use of a modified Hill equation (Hill, 1910). Note that other equations are sometimes used for specific cases, e.g. when multiphasic relationships are expected. Several tools for Hill equation-based dose-response analysis exist, varying in availability, analysis method and statistics. However, most tools are either commercial or lack graphical user interfaces, *de facto* impeding the adoption by biologists and clinicians. The programme most commonly cited for dose-response analyses is the commercial software GraphPad Prism (https://www.graphpad.com/scientific-software/prism/). A drawback of GraphPad is that it is commercial and, despite an extensive user guide and support page, the software remains a black box to the user as to the code and algorithms employed to analyse data. Moreover, GraphPad lacks a direct connection to a data storage system, requiring the user to manually annotate and import the relevant data. Here, we therefore introduce DoRes, a free, open-source and automated module for dose-response analysis in the CellMissy software framework. CellMissy is an open-source, cross-platform data management and analysis system for cell migration data that simplifies and automates data management, storage, quality control and analysis (Masuzzo *et al.*, 2013, 2017). CellMissy allows for the storage and analysis of both collective and single-cell migration data. The DoRes module is included in CellMissy from version 1.2, and is designed to handle both cell migration-specific data, as well as more generic dose-response data.

## 2 Tool description

DoRes allows the user to complement a traditional migration analysis in CellMissy with dose-response capabilities, but also allows dose-response analysis on generic (non-migration) data sets (see Supplementary Material S1). This choice in input data is of functional relevance, as it allows processing of e.g. toxicity or proliferative effects of potential drugs, alongside their migratory effects, all in a single tool. DoRes takes as input either a collective cell migration experiment as stored in CellMissy’s relational database, or a tabular file with compound doses and measured responses. When migration data is loaded from the CellMissy database, the annotations included in these data allow DoRes to identify all time points and conditions, which in turn enables fully automated analysis. Moreover, DoRes will also ignore any data previously excluded by CellMissy’s quality control. The default DoRes results interface provides plots for visual inspection and a table of estimated parameter values and statistics (Supplementary Material S2). If the data were obtained from the CellMissy database, an annotated plate view with conditions is added to the interface (Supplementary Material S3). In the data table, DoRes provides the best-fit values of the modified Hill equation parameters, their standard error and 95% confidence interval as well as the *R*^2^ of the fitted curve. If more than one treatment is applied, the user can choose the conditions to analyse, and different normalization strategies and parameter constraints (e.g. for known minimum or maximum response) can be applied to the curve fitting. Moreover, DoRes bundles all results in a detailed analysis report, which provides a general overview of the experiment, all plots and statistics and information on any applied normalization (see Supplementary Material S4). To demonstrate the capabilities of DoRes, we have analysed the ‘glymet’ dataset from the free R package drc (Ritz *et al.*, 2015, https://cran.r-project.org/web/packages/drc/drc.pdf) (see Supplementary Material S5). As shown in Table 1, DoRes produces results that are closely comparable to those of GraphPad Prism. We have also validated DoRes by performing and analysing collective cell migration experiments (see Supplementary Material S6). Importantly, this illustrates that DoRes handles high variance data better than GraphPad Prism, which in this case struggles to provide confidence intervals for key parameters. DoRes thus qualitatively compares with GraphPad in analysis properties but has the extra benefits of being open-source, of being integrated in the CellMissy data storage system, and of better handling of high variance data.

**Table 1.** Comparison of the statistics of the drc glymet dataset analysis with DoRes and GraphPad

	DoRes	GraphPad prism
Best-fit value		
Bottom	0.027 (0.101)	−0.02868 (0.1177)
Top	1.595 (0.035)	1.616 (0.04544)
Hill slope	−1.289 (0.172)	−1.188 (0.1752)
LogEC50	5.171 (0.063)	5.183 (0.05953)
EC50	14.8E+04	15.2E+04
*R*²	0.93	0.9326
95% CI		
Bottom	−0.171 to 0.225	−0.4785 to 0.1625
Top	1.527–1.663	1.536–1.752
Hill slope	−1.626 to −0.952	−1.618 to −0.7988
LogEC50	5.063–5.28	5.082–5.386
EC50	11.57E04–19.04E04	12.06E04–24.31E04

*Note*: Standard errors are between brackets.

Due to the need to preformat and import the relevant data by hand, the current use of generic, and often commercial dose-response tools is suboptimal (see Supplementary Material S7 for a comparison of GUI-based, freely available software for dose-response). Because DoRes is integrated in the CellMissy software, it can automatically retrieve all relevant experimental data and metadata from the CellMissy database, obviating data manipulation by the user. DoRes fully meets the capabilities of existing tools, is free and open-source and inherits its platform-independence from CellMissy.

## Supplementary Material

Supplementary Information
