# Supplementary material for: DoRes within CellMissy: dose-response analysis on cell migration and related data
Source: Bioinformatics. 2018 Jul 25;35(4):696–7. doi: 10.1093/bioinformatics/bty634 (PMC6378935; doi:10.1093/bioinformatics/bty634)
Supplement: Supplementary Information [file bty634_supplementary_information.docx]

**Supplementary Information for**

**DoRes within CellMissy: dose-response analysis on cell migration and related data**

Gwendolien Sergeant^1,2,*^, Lennart Martens^1,2^, Marleen Van Troys^2^ and Paola Masuzzo^1,2^[[1]](#footnote-1)^^

^1^ VIB-UGent Center for Medical Biotechnology, A. Baertsoenkaai 3, B-9000, Ghent, Belgium.

^2^ Department of Biochemistry, Faculty of Medicine and Health Sciences, Ghent University,
A. Baertsoenkaai 3, B-9000, Ghent, Belgium

**S1 - General tool functionality**

DoRes is a module for dose-response analysis, developed for the data management and analysis system CellMissy (Masuzzo et al., 2013, 2017). As shown in **Figure S1**, dose-response analysis can be performed on two types of input within DoRes. As generic input, a tabular file can be imported, containing doses and responses of any type or any measured property the user wishes to analyse. The second starting point of the new DoRes module is situated downstream of the collective migration analysis regression step in CellMissy, where cell sheet velocities are extracted and statistically compared across conditions. Dose-response analysis can at this point be carried out using the treatment concentrations and the extracted velocities. Note that DoRes in this case works on data that are stored together with detailed information on the experimental design (meta-data) in the CellMissy relational database. In both input types, multiple replicate values per concentration are possible, conform the classical set-up of a biological experiment.

DoRes fits the data to a modified Hill Equation, enabling furthermore the normalization of the responses and constraint of the parameters. This equation has four parameters: Bottom, Top, Hill slope and LogEC50, with the last the crucial parameter for assessing the biological effect of a tested compound. Note that other equations are sometimes used for specific cases, e.g. when multiphasic relationships are expected.

(1)


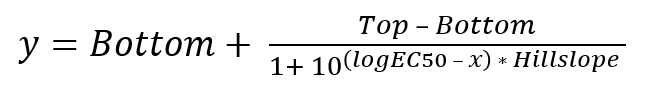
**Equation 1. Standard dose-response curve-fitting: modified Hill equation.** x is logarithm of tested compound concentration; y is measured effect. Top and Bottom are maximal and minimal response, respectively; EC50 is compound concentration with 50% measured effect.

**
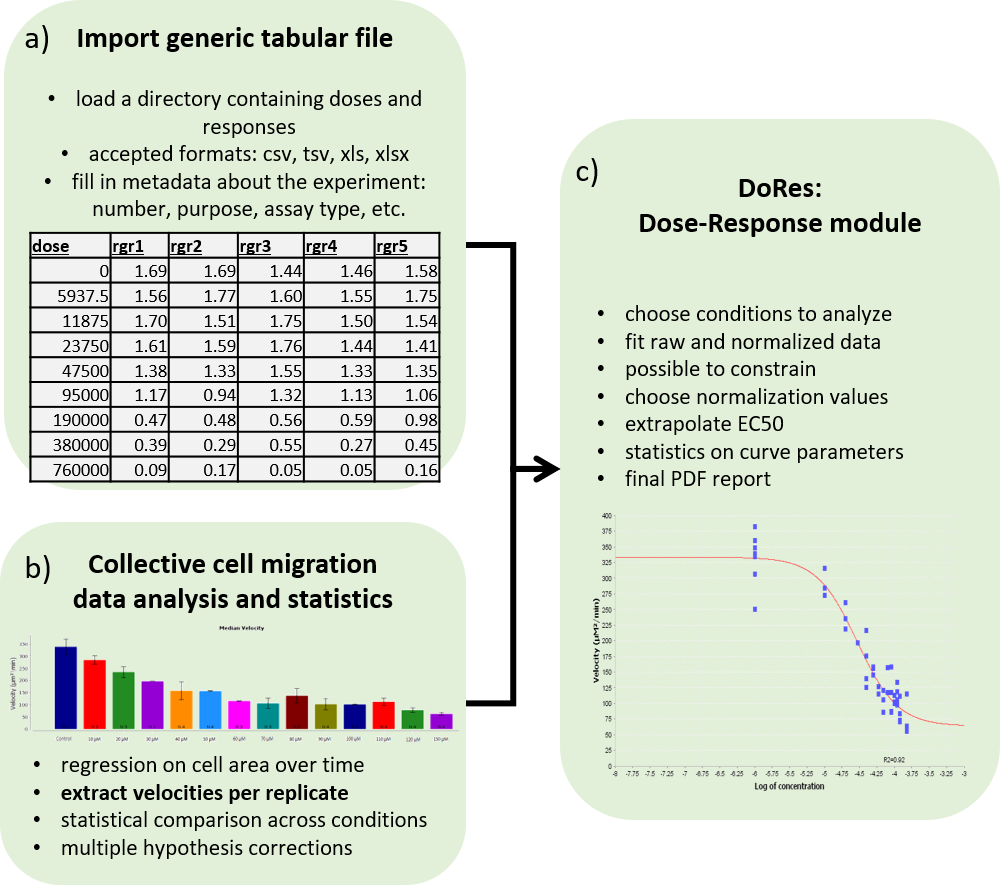
**

**Figure S1 Overview of DoRes**. The module accepts as input either **(a)** a generic table with doses and responses (for the generic data used in this article, see Table S1) or **(b)** the results from CellMissy’s extracted migration velocities coupled to the tested conditions or replicates within conditions. The input data is analysed by DoRes **(c)**.

**S2 - Results view for generic data analysis**


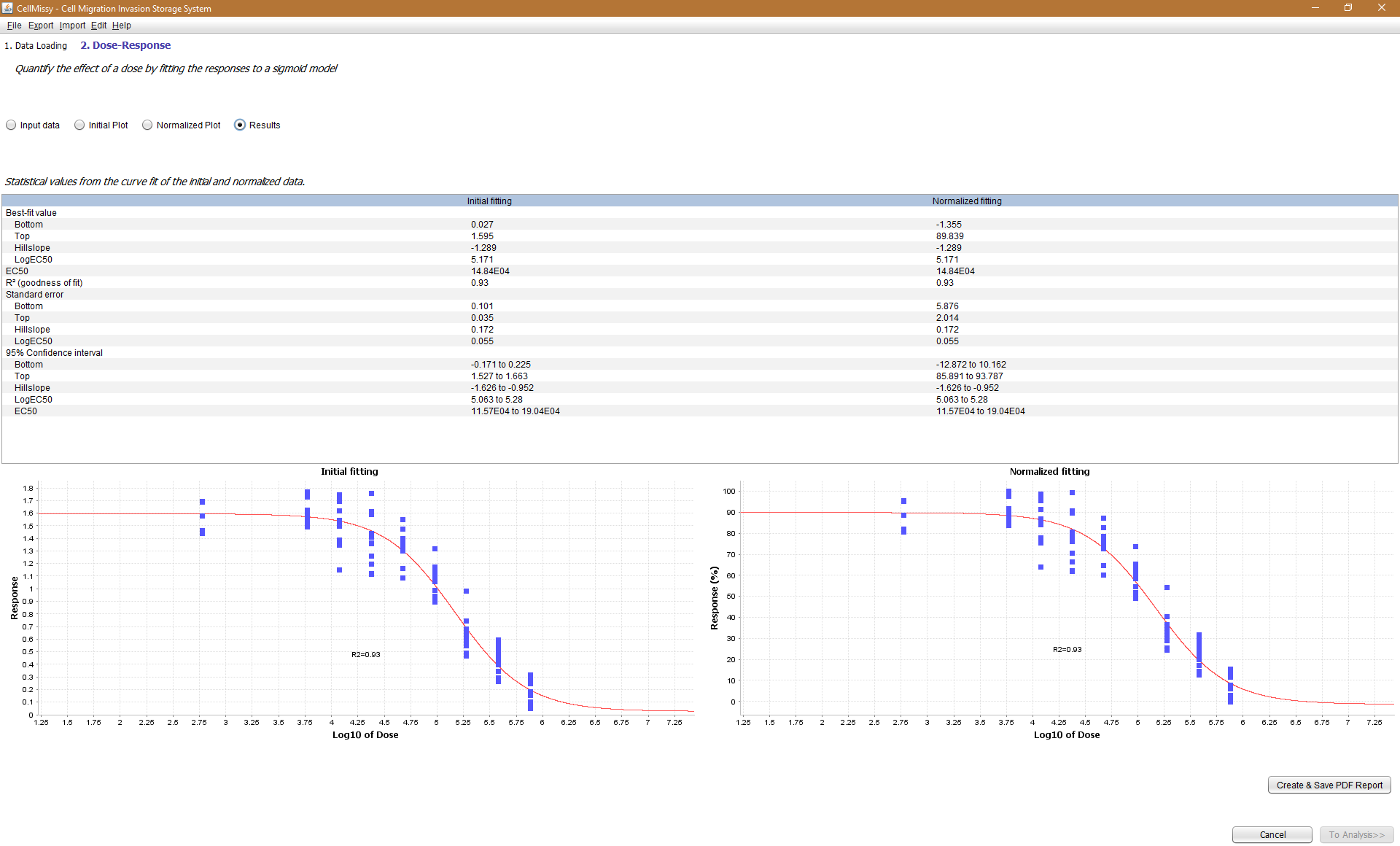


**Figure S2 Example of the results tab when analysing generic data**. The dataset shown here is the ‘glymet’ dataset from the drc package. More information on this dataset can be found in Supplementary data S5 and Table 2 in the main article.

**S3 - Results view for cell migration data analysis**


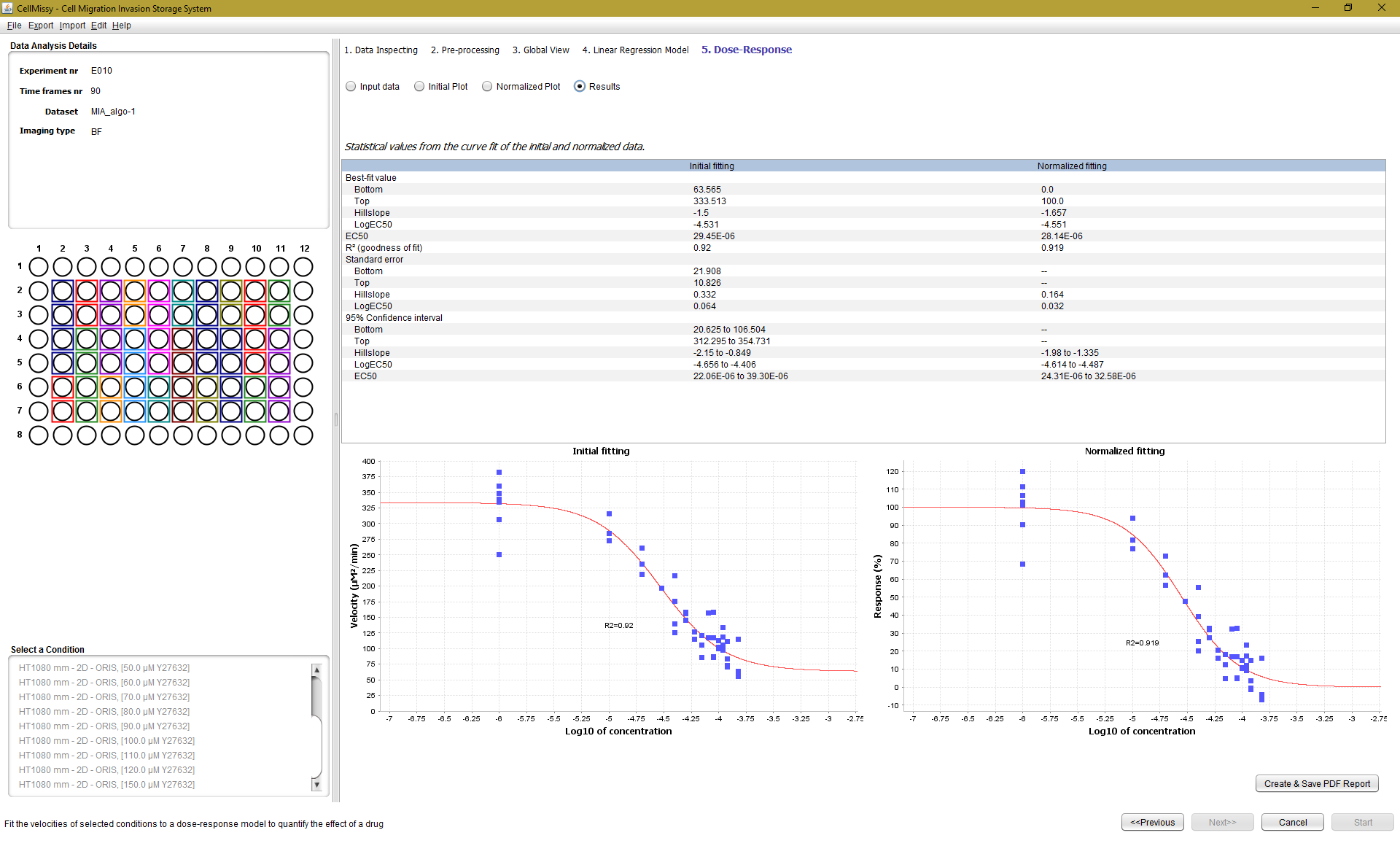


**Figure S3 Example of the results tab when analysing generic data**. The dataset in which the fibrosarcoma cell line HT1080 is treated with different doses of the Rock inhibitor Y27632 (see methods) used here is the same as in **Supplementary data S4**, **Figure S5** and **Table S2**.

**S4 - Example of a DoRes dose-response analysis report**

The same experimental data is also represented in **Supplementary data S3**, **Figure S5** and **Table S2**.

**CellMissy - DOSE RESPONSE ANALYSIS REPORT - EXPERIMENT E010 - PROJECT P027**

DATASET: MIA_algo-1

NUMBER OF BIOLOGICAL CONDITIONS: 14

DRUG ANALYSED: Y27632

**ANALYSIS GROUP SUMMARY**

| **DRUG**  **CONCENTRATION** | **# TECHNICAL**  **REPLICATES** | **TECHNICAL**  **REPLICATES EXCLUDED?** | **LOWEST VELOCITY** | **HIGHEST VELOCITY** | **MEDIAN VELOCITY** |
| --- | --- | --- | --- | --- | --- |
| Control | 8 | YES, 1 | 249.802 | 382.301 | 338.763 |
| 10.0 µM | 4 | YES, 1 | 272.14 | 315.325 | 284.368 |
| 20.0 µM | 4 | YES, 1 | 219.162 | 260.925 | 234.621 |
| 30.0 µM | 4 | YES, 3 | 196.737 | 196.737 | 196.737 |
| 40.0 µM | 4 | NO | 125.756 | 216.765 | 157.475 |
| 50.0 µM | 4 | NO | 144.93 | 158.116 | 156.344 |
| 60.0 µM | 4 | YES, 1 | 115.158 | 126.775 | 115.5 |
| 70.0 µM | 4 | YES, 1 | 86.079 | 120.344 | 105.988 |
| 80.0 µM | 4 | YES, 2 | 116.984 | 157.046 | 137.015 |
| 90.0 µM | 4 | NO | 85.931 | 157.826 | 102.12 |
| 100.0 µM | 4 | NO | 100.33 | 112.374 | 101.4 |
| 110.0 µM | 4 | NO | 97.231 | 133.59 | 112.06 |
| 120.0 µM | 4 | NO | 70.771 | 111.94 | 77.984 |
| 150.0 µM | 4 | NO | 55.911 | 114.922 | 62.192 |

**INITIAL FIT**

CONSTRAINED PARAMETERS: BOTTOM = -- TOP = --

R SQUARED (GOODNESS OF FIT) = 0.92

| **Parameter** | **Best-fit value** | **Standard Error** | **95% Confidence Interval** |
| --- | --- | --- | --- |
| Bottom | 63.565 | 21.908 | 20.625 to 106.504 |
| Top | 333.513 | 10.826 | 312.295 to 354.731 |
| LogEC50 | -4.531 | 0.064 | -4.656 to -4.406 |
| Hillslope | -1.5 | 0.332 | -2.15 to -0.849 |
| EC50 | 2.94E-05 | -- | 2.21E-05 to 3.93E-05 |


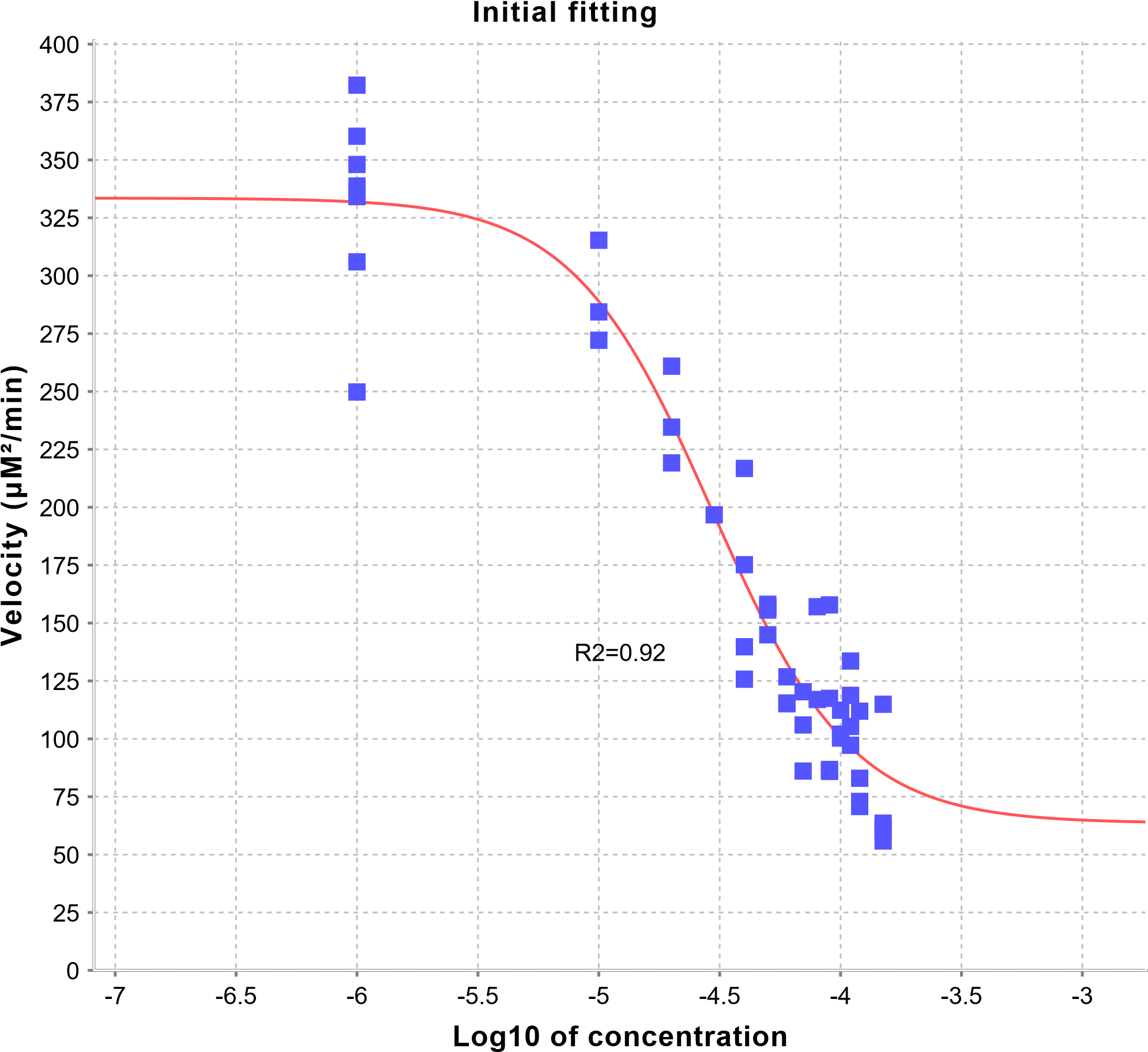


**NORMALIZED FIT**

NORMALIZATION: 0% = 73.8 (Smallest Mean Value) 100% = 331.32 (Largest Mean Value) CONSTRAINED PARAMETERS: BOTTOM = 0.0 TOP = 100.0

R SQUARED (GOODNESS OF FIT) = 0.919

| **Parameter** | **Best-fit value** | **Standard Error** | **95% Confidence Interval** |
| --- | --- | --- | --- |
| Bottom | 0.0 | -- | -- |
| Top | 100.0 | -- | -- |
| LogEC50 | -4.551 | 0.032 | -4.614 to -4.487 |
| Hillslope | -1.657 | 0.164 | -1.98 to -1.335 |
| EC50 | 2.81E-05 | -- | 2.43E-05 to 3.26E-05 |


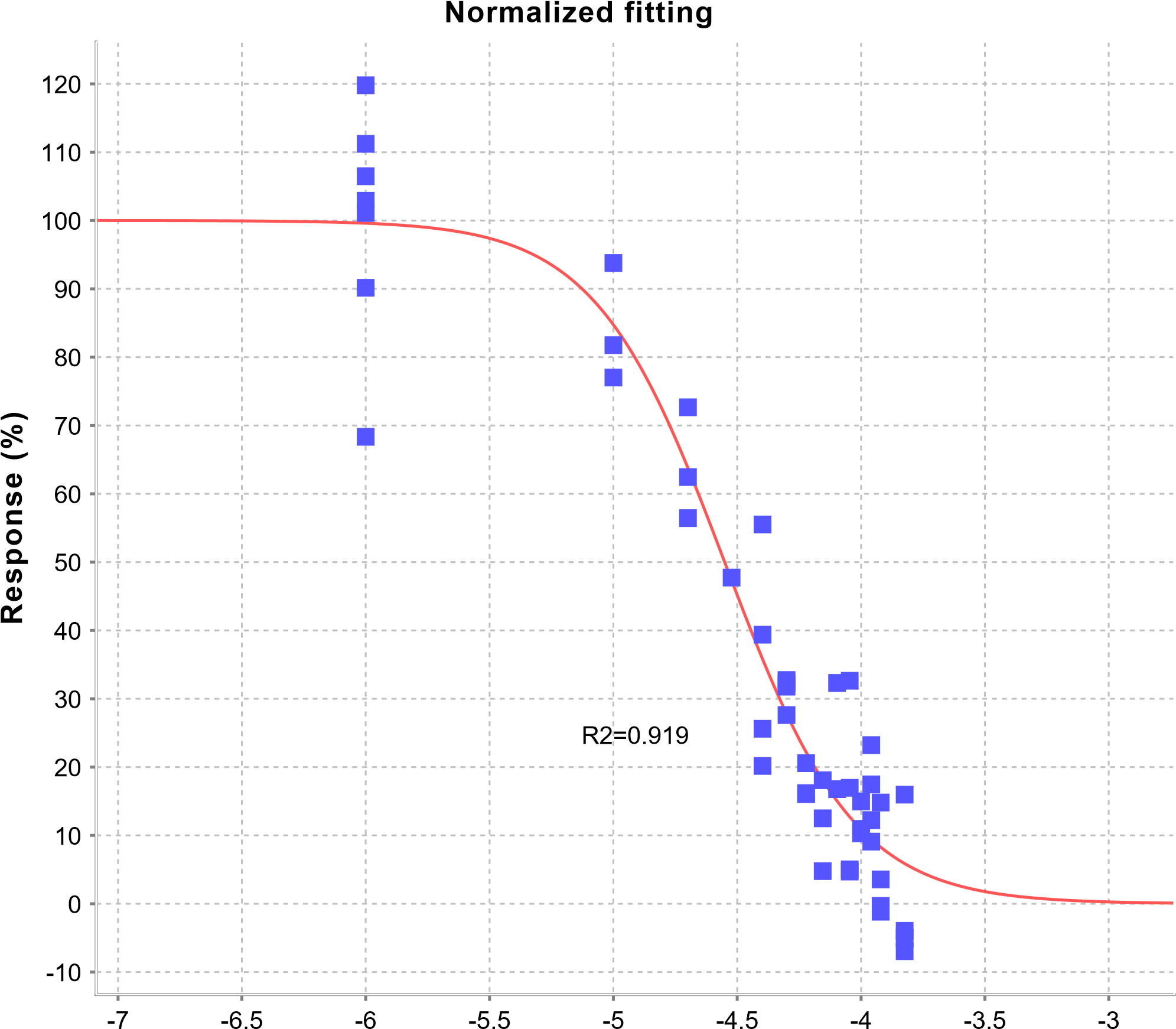


**Log10 of concentration**

**S5 - Generic dose-response files**

DoRes has the ability to import and analyse generic tables containing doses and responses, even though CellMissy is focused on cell migration data. This option can be very useful for proliferation experiments, which are often performed alongside migration assays when testing compounds. To demonstrate this functionality, we have opted to use a freely available dose-response dataset, namely the ‘glymet’ dataset from a free R package called ‘drc’ (for a full description of the contents of this package, see: <https://cran.r-project.org/web/packages/drc/drc.pdf>; the dataset is the output of measuring algae growth rate in the absence or presence of herbicides). Table S1 shows the entire dose response dataset with nine tested concentrations and 5-14 replicates per concentration. Figure S2 demonstrates the fitted curve in DoRes. Statistics for this graph can be found in the main article (Table 2).

**Table S1 - Input data: glymet dataset from the drc package.**

| **Dose** | **rgr1** | **rgr2** | **rgr3** | **rgr4** | **rgr5** | **rgr6** | **rgr7** | **rgr8** | **rgr9** | **rgr10** | **rgr11** | **rgr12** | **rgr13** | **rgr14** |
| --- | --- | --- | --- | --- | --- | --- | --- | --- | --- | --- | --- | --- | --- | --- |
| 0 | 1.69 | 1.69 | 1.44 | 1.46 | 1.58 |  |  |  |  |  |  |  |  |  |
| 5937.5 | 1.56 | 1.77 | 1.60 | 1.55 | 1.75 | 1.57 | 1.76 | 1.59 | 1.73 | 1.49 | 1.52 | 1.62 |  |  |
| 11875 | 1.70 | 1.51 | 1.75 | 1.50 | 1.54 | 1.37 | 1.62 | 1.39 | 1.70 | 1.52 | 1.69 | 1.35 | 1.15 |  |
| 23750 | 1.61 | 1.59 | 1.76 | 1.44 | 1.41 | 1.36 | 1.36 | 1.26 | 1.43 | 1.12 | 1.41 | 1.12 | 1.19 |  |
| 47500 | 1.38 | 1.33 | 1.55 | 1.33 | 1.35 | 1.16 | 1.37 | 1.47 | 1.39 | 1.30 | 1.40 | 1.35 | 1.37 | 1.08 |
| 95000 | 1.17 | 0.94 | 1.32 | 1.13 | 1.06 | 0.90 | 0.99 | 0.90 | 1.06 | 0.92 | 1.15 | 1.09 | 1.07 | 0.90 |
| 190000 | 0.47 | 0.48 | 0.56 | 0.59 | 0.98 | 0.75 | 0.63 | 0.61 | 0.55 | 0.49 | 0.68 | 0.68 | 0.64 | 0.57 |
| 380000 | 0.39 | 0.29 | 0.55 | 0.27 | 0.45 | 0.49 | 0.42 | 0.51 | 0.35 | 0.29 | 0.51 | 0.51 | 0.59 | 0.57 |
| 760000 | 0.09 | 0.17 | 0.05 | 0.05 | 0.16 | 0.16 | 0.08 | 0.16 | 0.10 | 0.18 | 0.31 | 0.28 | 0.27 | 0.25 |

Rgr: relative growth rate. Changes from original: additional data not pertaining to dose or response have been removed, values are rounded up to two decimals and reformatted so that replicates are shown in separate columns.


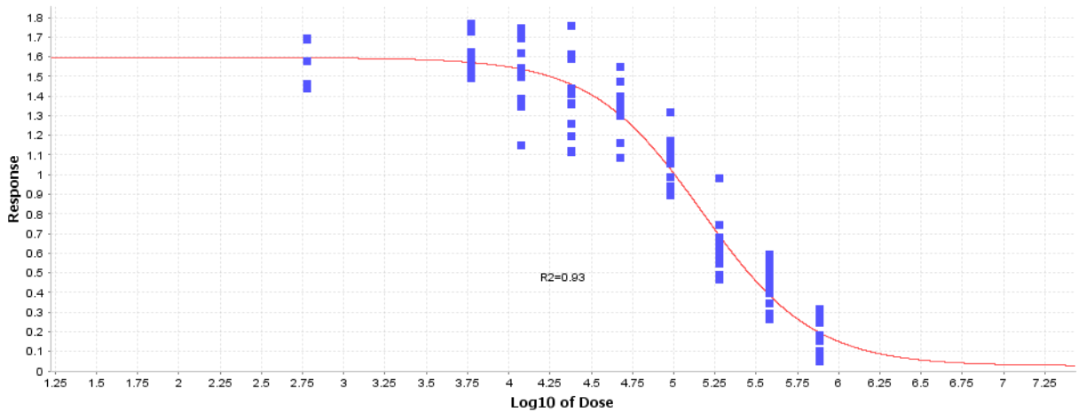


**Figure S4: DoRes graph of the glymet dataset.** The original doses have been log-transformed to allow for better visual spreading. Control dose is automatically set to the lowest log10 dose minus one. Each replicate (in blue) is considered for the fitting (in red).

**S6 - Investigating cell migration experiments.**

Effect on cell migration was tested in a cell-zone exclusion assay (see Methods section S8 for detail). For these migration experiments, the responses are velocities extracted from linear regression on the increase in area occupied by the migrating cell population over time, in the absence or presence of a range of drug. We here present dose-response data in which HT1080 cells have either been treated with a migration inhibiting compound (Figure S5) or a compound that stimulates migration (Figure S6). In the first case, DoRes fitting and results are very similar to the ones produced by GraphPad (see Table S2). It is important to note that the confidence intervals cannot be strictly compared with GraphPad Prism, because the commercial software’s newest version (version 7) now computes profile likelihood intervals. These are skewed confidence intervals, which DoRes does not calculate. As reported on the GraphPad curve fitting guide, this only does a better job “for some parameters in some models”. (see <http://www.graphpad.com/guides/prism/7/curve-fitting/reg_how_confidence_intervals_are_c.htm>) Indeed, on high-variance data points as presented in Figure S6, GraphPad is not able to calculate any confidence intervals (see Table S3).


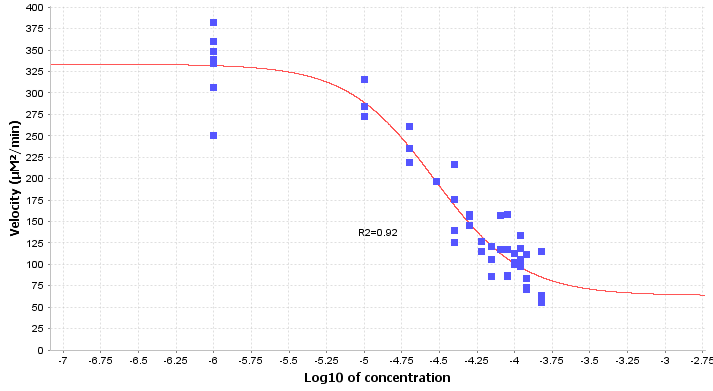


**Figure S5. DoRes graphs of cell migration experiments with low-variance data.**
Experiment with migration-inhibiting compound: HT1080 cells treated with Y27632.

**Table S2. Statistics of Figure S5.**

Standard errors are between brackets.

|  | **DoRes** | **GraphPad Prism** |
| --- | --- | --- |
| **Best-fit value** |  |  |
| Bottom | 63.565 (21.908) | 63.53 (21.93) |
| Top | 333.513 (10.826) | 333.5 (10.83) |
| Hill slope | -1.5 (0.332) | -1.499 (0.3319) |
| LogEC50 | -4.531 (0.064) | -4.531 (0.064) |
| EC50 | 29.5E-06 | 29.5E-06 |
| **R²** | 0.92 | 0.9197 |
| **95% Confidence interval** |  |  |
| Bottom | 20.625 to 106.504 | -40.82 to 95.13 |
| Top | 312.295 to 354.731 | 312.7 to 358.6 |
| Hill slope | -2.15 to -0.849 | -2.309 to -0.83 |
| LogEC50 | -4.656 to -4.406 | -4.65 to -4.278 |
| EC50 | 22.06E-06 to 39.30E-06 | 22.38E-06 to 52.72E-06 |


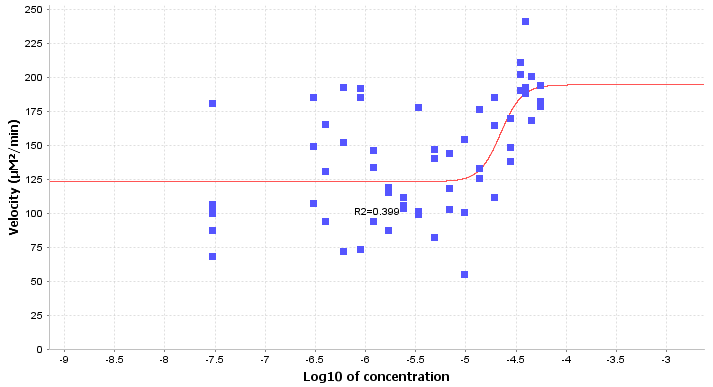


**Figure S6. DoRes graphs of cell migration experiments with high-variance data.**
Experiment with migration-stimulating compound: HT1080 cells treated with LY294002.

**Table S3. Statistics of Figure S6.**

Standard errors are between brackets.

|  | **DoRes** | **GraphPad Prism** |
| --- | --- | --- |
| **Best-fit value** |  |  |
| Bottom | 123.257 (5.662) | 123.3 (5.661) |
| Top | 194.528 (19.946) | 194.6 (19.96) |
| Hill slope | 4.101 (3.848) | 4.103 (3.851) |
| LogEC50 | -4.653 (0.124) | -4.653 (0.1235) |
| EC50 | 22.2E-06 | 22.2E-06 |
| **R²** | 0.399 | 0.3992 |
| **95% Confidence interval** |  |  |
| Bottom | 112.16 to 134.354 | 111.8 to 135.6 |
| Top | 155.432 to 233.623 | 170 to +infinity |
| Hill slope | -3.442 to 11.642 | ??? to 37.97 |
| LogEC50 | -4.896 to -4.411 | -4.904 to ??? |
| EC50 | 12.71E-06 to 38.82E-06 | 12.49E-06 to ??? |

#### S7 – Comparing DoRes with other dose-response tools

The program most commonly cited for dose-response analyses is the commercial software GraphPad Prism (<https://www.graphpad.com/scientific-software/prism/>). As a result, GraphPad has been used as the validation standard for other dose-response packages (Gadagkar and Call, 2015). Compared to the freely available tool HEPB (Table S4) for generic dose-response curve fitting (Gadagkar and Call, 2015), DoRes has the important advantage of applying fewer constraints. Indeed, HEPB automatically constrains the values of the minimum and maximum response plateaus, which is rarely the case in a biological study. DoRes also distinguishes itself from HEPB by providing statistics for all estimated Hill equation parameters, and by offering normalization options.

The second generic dose-response curve fitting software listed in Table S4 is Dr Fit, a free tool that can handle multiphasic models (Di Veroli et al., 2015), in which more than one point of inflection is found, or which combines agonist and antagonist effects. Dr. Fit achieves this by fitting a model to each of four predefined sets of phases (one monophasic, two biphasic, and one triphasic model) and then uses a ranking test to choose the optimal model. As such, Dr Fit provides EC50 and model parameters for all four accounted combinations of (multi-)phasic relationships, but does not calculate any detailed statistics on the models themselves like DoRes; rather, statistics are only calculated for comparison between the different models. Moreover, Dr. Fit, despite being free, is not open source.

The final two tools listed in Table S4, CABAS and IncucyteDRC, are specifically developed for cell biology applications. CABAS is specifically created to study chromosome aberration dosimetry and radiation effects (Deperas et al., 2007), and uses a linear-quadratic model with a maximum of twenty data points as input. DoRes has no such maximum data point limit. IncucyteDRC, provides a workflow for cell proliferation studies using the IncuCyte ZOOM timelapse microscope (Chapman et al., 2016). IncucyteDRC is a Shiny web interface on top of the drc R package to fit curves, and while capable of rudimentary dose-response curve fitting, the authors explicitly recommend exporting data to GraphPad for this task (Chapman et al., 2016). Moreover, unlike DoRes, IncucyteDRC also does not provide any statistics for the obtained Hill equation parameter values.

**Table S4.** Comparison of DoRes with other dose-response analysis software, focusing predominantly on free tools.

HE: Hill equation, GOF: goodness of fit, SE: standard error, CI: confidence interval, PB: prediction band, AIC: Akaike information criterion, BIC: Bayesian information criterion

| **Software** | **Fitting**  **equation** | **Flexible**  **parameter constraining** | **Number of estimated parameters** | **Statistics** | **Not limited to specific analysis question** | **Free** | **Open Source** | **Unique traits** | **Reference** |
| --- | --- | --- | --- | --- | --- | --- | --- | --- | --- |
| **DoRes** | HE | + | 4 - #constrained | GOF, SE, CI | + | + | + | Backend database, supports experimental setup |  |
| **GraphPad Prism** | Weighted HE | + | 5 - #constrained | GOF, SE, CI | + | - | - | Extensive analysis possibilities |  |
| **HEPB** | HE | - | 2 | PB | + | + | - |  | *(Gadagkar and Call, 2015)* |
| **Dr Fit** | HE + multiphasic | - | 3 * amount of phases | Chi², GOF, AIC, BIC | + | + | - | Fits multiphasic relationships | *(Di Veroli et al., 2015)* |
| **CABAS** | Linear –  quadratic | - | 3 | GOF, SE | - | + | - | Performs dose and uncertainty calculation | *(Deperas et al., 2007)* |
| **IncucyteDRC** | Log – logistic. Utilizes the drc package | - | 4 | Depends on analysis package | - | + | + | Follows experimental workflow, allows data export for analysis | *(Chapman et al., 2016)* |

#### S8 – Materials & Methods

**1. Cell cultures**

The cell line used was HT1080 (fibrosarcoma, ATCC number CCL-121™). Cell growth medium was Dulbecco’s modified Eagle’s medium (DMEM) with GlutaMax, 4.5g/l D-glucose and 1mM Na-Pyruvate (Gibco® BRL, UK) with additional 10% fetal bovine serum (FBS) (Gibco® BRL, UK) and 1% penicilin-streptomycin (Gibco® BRL, UK). Cells were kept in an incubator at 37°C in a 5% CO_2_ atmosphere.

**2. Cell migration assays**

Migration was tested using the ORIS^TM^ cell migration protocol (Platypus Technologies), a cell exclusion zone assay. Cells were seeded in a 96-well plate on a coating of monomeric rat tail collagen type I (BD Biosciences, US) (concentration 40μg/ml, 100 μl added to wells, incubated 1 hour at room temperature). Cell seeding (47500 cells per well) was done 24 h prior to start of the migration around stoppers (Oris™ Cell Seeding Stoppers, Platypus Technologies) and resulted in a confluent cell layer around the central zone. Before starting the imaging, the stoppers were removed and 200μl assay medium with the indicated drug concentration was added to the cells. Assay medium contained only 1% FBS. Phase contrast time-lapse imaging was performed for 36 hours with 20 minute intervals on a CellM Life Cell Imaging System comprising an Olympus iX71 microscope. Image processing (segmentation of the area covered by the migrating cell population in time) was done using CELLMIA software (Van Troys et al, 2018).

**3. Assay drugs and treatments**

LY294002 is a PI3K-inhibitor and a component of the InhibitorSelect™ EGFR Signaling Pathway Inhibitor Panel (Calbiochem® Merck©). Stock solutions of the drugs (48796.5 μM) were made in dimethylsulfoxide (DMSO) (SIGMA®). Final DMSO concentration in all samples was 0.11%.

Y27632 is a ROCK inhibitor (Sigma-Aldrich®). Stock solutions (59000 μM) were made in water.

**4. Data**

Drc is included in RStudio. For analysis with DoRes, we have simplified the datasets to only contain doses and responses. Three datasets have been included in the CellMissy files as example dose-response datasets. These datasets have been chosen according to usability (one treatment, high amount of doses and replicates per dose). We have also included three migration dose-response data files, two of which are featured in this supplementary data.

**References**

Chapman, P.J., et al. (2016). IncucyteDRC: An R package for the dose response analy-sis of live cell imaging data. F1000Res 5, 962.

Deperas, J., et al. (2007). CABAS: a freely available PC program for fitting calibration curves in chromosome aberration dosimetry. Radiat Prot Dosimetry 124, 115–123.

Di Veroli, G.Y., et al. (2015). An automated fitting procedure and software for dose-response curves with multiphasic features. Sci Rep 5, 14701.

Gadagkar, S.R., and Call, G.B. (2015). Computational tools for fitting the Hill equation to dose-response curves. J Pharmacol Toxicol Methods 71, 68–76.

Masuzzo, P., et al. (2013). CellMissy: a tool for management, storage and analysis of cell migration data produced in wound healing-like assays. Bioinformatics 29, 2661–2663.

Masuzzo, P., et al. (2017). An end-to-end software solution for the analysis of high-throughput single-cell migration data. Sci Rep 7, 42383.

Van Troys, M, et al. Analysis of Invasion Dynamics of Matrix-Embedded Cells in a Multisample Format. Book chapter in: Cell Migration – Methods and Protocols. © 2018 Alexis Gautreau (Ed.). Book series: Methods in Molecular Biology; series volume 1749. DOI 10.1007/978-1-4939-7701-7 Publisher Humana Press

1. * To whom correspondence should be addressed [↑](#footnote-ref-1)
